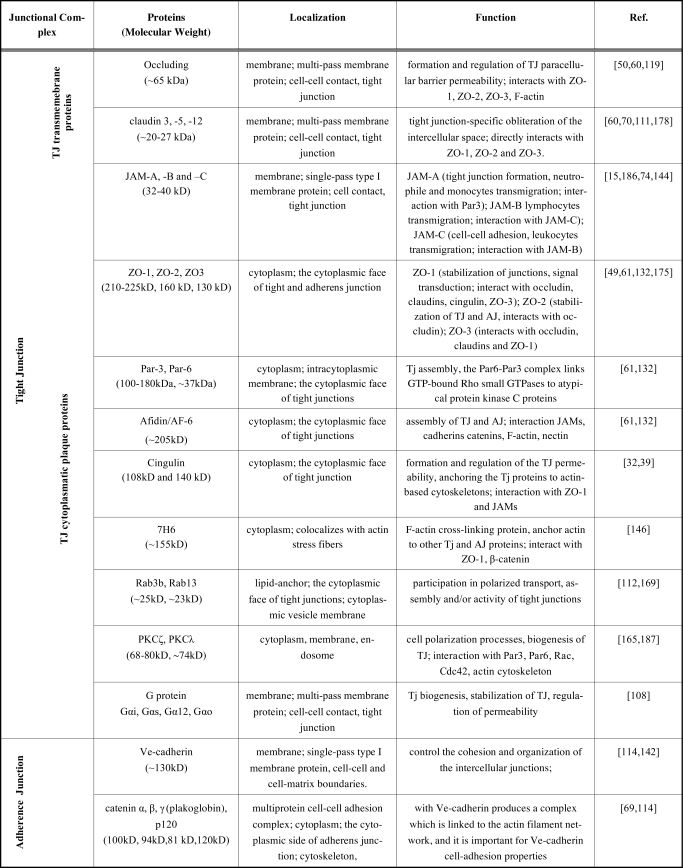# Brain Endothelial Cell-Cell Junctions: How to “Open” the Blood Brain Barrier

**DOI:** 10.2174/157015908785777210

**Published:** 2008-09

**Authors:** Svetlana M Stamatovic, Richard F Keep, Anuska V Andjelkovic

**Affiliations:** 1 Department of Pathology, University of Michigan, Ann Arbor MI 48109, USA; 2 Department of Neurosurgery, University of Michigan, Ann Arbor MI 48109, USA; 3 Department of Molecular and Integrative Physiology, University of Michigan, Ann Arbor MI 48109, USA

## Abstract

The blood-brain barrier (BBB) is a highly specialized structural and biochemical barrier that regulates the entry of blood-borne molecules into brain, and preserves ionic homeostasis within the brain microenvironment. BBB properties are primarily determined by junctional complexes between the cerebral endothelial cells. These complexes are comprised of tight and adherens junctions. Such restrictive angioarchitecture at the BBB reduces paracellular diffusion, while minimal vesicle transport activity in brain endothelial cells limits transcellular transport. Under normal conditions, this largely prevents the extravasation of large and small solutes (unless specific transporters are present) and prevents migration of any type of blood-borne cell. However, this is changed in many pathological conditions. There, BBB disruption (“opening”) can lead to increased paracellular permeability, allowing entry of leukocytes into brain tissue, but also contributing to edema formation. In parallel, there are changes in the endothelial pinocytotic vesicular system resulting in the uptake and transfer of fluid and macromolecules into brain parenchyma. This review highlights the route and possible factors involved in BBB disruption in a variety of neuropathological disorders (e.g. CNS inflammation, Alzheimer’s disease, Parkinson’s disease, epilepsy). It also summarizes proposed signal transduction pathways that may be involved in BBB “opening”.

## INTRODUCTION

“Good fences make good neighbors.” The blood-brain barrier (BBB) is a highly specialized structural, transport and biochemical (enzymatic) barrier, that mainly consists of microvascular endothelial cells and overlying astrocytic foot processes. It regulates the entry of compounds and cells between blood and brain and, thus, has a fundamental role in brain homeostasis. It also, though, forms a route of communication between circulating blood and underlying brain tissues [125,142,173]. Much of the structural barrier is due to the presence of tight junctions between the cerebral endothelial cells that limit paracellular diffusion. Those junctions, how they are regulated, how they are affected by disease states and how they can be manipulated therapeutically is the main focus of this review. Other reviews discuss other BBB properties (e.g. transport and enzymatic barrier properties) in depth.

## BBB STRUCTURE: THE JUNCTIONAL COMPLEXES

BBB properties are primarily determined by endothelial junctional complexes consisting of tight junctions (TJ) and adherens junctions (AJ). It is generally accepted that TJ seal the interendothelial cleft forming a continuous blood vessel, while the AJ are important for initiating and maintaining endothelial cell-cell contact [60,142]. The tight junctions between BBB endothelial cells lead to high endothelial electrical resistance and low paracellular permeability. The electrical resistance is in the range of 1500-2000 Ω.cm^2^ (pial vessels) compared to 3-33 Ω.cm^2^ in other tissues [27,40].

**Fig. (1):**
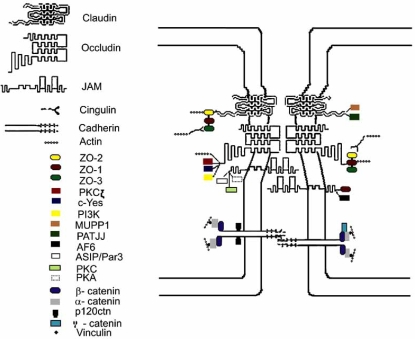
Structural features of the brain endothelial junctional complex.

TJ and AJ are composed of transmembrane proteins and cytoplasmic plaque proteins (Fig. **1**, Table **1**). The former proteins physically associate with their counterparts on the plasma membrane of adjacent cells, whereas the latter provide a link between transmembrane TJ/AJ proteins and the actin cytoskeleton but also participate in intracellular signaling [60,142]. 

Transmembrane proteins of the TJ include occludin, claudins (for example, claudin-3, -5, -12) and junctional adhesion molecule (JAM)-A, JAM-B and JAM-C [32,60,103]. Occludin was one of the first TJ transmembrane proteins described. Structurally, occludin contains two equal extracellular loops (44 amino acids), four transmembrane domains and three cytoplasmic domains: one intracellular short turn, a small N-terminal domain and a long carboxyl (C-) terminal [60,92,119,150]. It is thought that both of the extracellular loops provide the gate and fence-like structure of TJs, although the function of first extracellular loop is mostly involved in intercellular adhesion, while second loop mostly affects transendothelial electrical resistance [50,119]. The C-terminal domain (150 amino acids) of occludin is remarkably conserved between species and it is predicted to form a typical α-helical coiled-coil structure [92,119]. Through this structure occludin can associate with ZO-1, ZO-2 and ZO-3, directly bind to F-actin, and interact with regulatory protein PKCζ, tyrosine kinase c-Yes, PI3K and gap junction component connexin 26 [4,35,85,129,165,168]. As well as being involved in the integration and function of occludin in the TJ complex, this part of the molecule plays a critical role in paracellular channel formation and in mediating the endocytosis and trafficking of occludin [73,112,168]. The N-terminal site is critical for occludin function as an adhesion molecule and a N-terminal occludin mutant may be unable to fully oligomerize and completely assemble in TJ complexes [148].

Claudins are principal barrier-forming proteins that belong to the PMP22/EMP/MP20/claudin family of proteins [for review see 14, 36, 60, 70]. Until now, twenty different claudins have been identified and each of them shows a unique pattern of tissue expression. All claudins have the same structural pattern: four membrane-spanning regions, two extracellular loops and two cytoplasmic termini: a very short internal N-terminal sequence (2-6 amino acids) and a longer internal C-terminal sequence (21-63 amino acids) [36,70]. The first extracellular loop (49-52 amino acids) influences paracellular charge selectivity, while the second extracellular loop (16-33 amino acids) is the receptor for a bacterial toxin [56,60,80,143]. The C-terminus possesses the binding site for the cytoplasmic proteins ZO-1, ZO-2, ZO-3, MUPP1, PATJ through a PDZ motif [56,64,143]. Claudins function in the TJ complex is to limit paracellular ion movement selectively and this produces the high electrical resistance of the barrier [104,178]. Brain endothelial cells possess the claudin-5 and claudin-12 and possibly some other claudins [112,160]. Claudins present in brain endothelial cells may form pores of variable size (~10Å) which may be involved in transjunctional movement of water [17,104, 121,178]. Claudin-5 deletion did not inhibit tight junction formation but did result in a size-selective increase in permeability [70,110].

JAMs (JAM-A, -B, -C) are members of the immunoglobulin superfamily [15,16]. These molecules structurally are composed of a single membrane spanning domain, an extracellular domain, an extracellular N-terminus, and a short cytoplasmic C-terminus [15,16,159,186]. The extracellular region of JAMs consists of two IgG-like domains. These are often subclassified as *C1*-, *C2-, V-* and *I*-type based on the variable and constant regions of antibodies [15,159,186]. The extracellular domain appears to be subject of glycosylation, although the function of this is still unknown [186]. The N-terminal loop can be dimerized. The short C-terminus cytoplasmic tail (40 amino acids) contains a PDZ binding domain which facilitates interactions with TJ associated scaffold proteins such as ZO-1, AF-6, ASIP/Par3, and cingulin [74,124]. The cytoplasmic tail also contains consensus phosphorylation sites that may serve as substrates for PKC and PKA [36,123,165]. New evidence also suggests that this cytoplasmic tail may be involved in targeting JAM to intercellular junctions. In general, JAMs are expressed at the intracellular junctions of endothelial and epithelial cells and display different patterns of homo- and heterophilic adhesion [15,86,144]. Homophilic interactions are with molecules on the opposite cell, forming JAM dimers that are part of tight junction structure [15,144]. Heterophilic interactions, however, can occur between different JAM family members as well as other adhesion molecules (e.g. integrins) and their function is still unknown [86]. Regarding the function JAMs at the level of the TJ complex, these molecules are involved in promoting the localization of ZO-1, AF6, CASK and occludin at points of cell contact and, *via* targeting Par3, are involved in establishment of cell polarity [16,74]. There is also convincing evidence supporting a role for the members of JAM family in the migration of leukocytes across endothelial junctions [7,16,53]. 

The TJ cytoplasmic plaque proteins are subdivided depending on whether they contain a PDZ motif. PDZ containing proteins include members of the family membrane-associated guanylate-kinase (MAGUK) homologues (ZO-1, -2, -3), partitioning-defective proteins (Par)3, Par6, afidin/Af-6, etc [60,61,132]. These proteins contain a PDZ motif (80-90 amino acids) on the carboxyl terminal that can mediate interactions with other proteins, transmembrane proteins or with PDZ motifs on other proteins [60,61,152]. The PDZ domain is critical for clustering and anchoring of transmembrane proteins. Most of these proteins contain multiple PDZ domains, so they may act as scaffolds that bring together cytoskeleton, signaling and integral proteins at specific regions of the plasma membrane [107,175]. For example, ZO-1 is thought to function as a multidomain scaffold that coordinates assembly of transmembrane and cytosolic proteins into TJ and/or regulates the activity of these proteins once they are assembled. ZO-1 binds to all three classes of TJ transmembrane proteins including occludin, claudins and CTX (IgG superfamily), but it also binds to 10 cytoplasmic proteins and various components of the cortical cytoskeleton [49,152,175]. In this case, ZO-1 is required for the normal kinetics of TJ assembly, for TJ specific localization and unique organization of transmembrane proteins [32,61].

PDZ lacking plaque proteins include as cingulin, 7H6, Rab13, ZONAB, AP-1, PKCζ, PKCλ, heterotrimeric G protein. These proteins exert a variety of functions at the TJ complex. Cingulin, for example, act as a cross link between TJ proteins (ZO-2, ZO-3, AF-6, JAM) and the actin-myosin cytoskeleton [32,39]. 7H6 mostly plays a role in TJ maintenance and maturation, while ZONAB is a transcription factor that regulates ErbB transcription and paracellular permeability [8,146]. Rab proteins (Rab13, Rab3b) have a role in docking and fusion of transport vesicles at the TJ complex [169]. PKCζ and PKCλ are involved in regulation of polarization as well as in TJ assembly, while G proteins (G_α-i0_, G_α-i2_, Gα12, G_αs_) co-immunoprecipitate with ZO-1 and play a role in accelerating TJ assembly and maintaining transendothelial electrical resistance [55,108,165,187].

Vascular endothelium cadherin (Ve-cadherin) is the major transmembrane protein of the endothelial AJ. A member of the large family of cadherins, it interacts homotypically in the presence of Ca^2+^. The AJ cytoplasmic plaque includes proteins of the catenin family (α, β, γ, p120) [15,114]. Ve-cadherin is an important determinant of microvascular integrity both *in vitro* and *in vivo* and together with catenin forms a complex that function as an early recognition mechanism between endothelial cells. [15,114,142,173]. It is believed that β- and γ-catenin link the cadherin to α-catenin which, in turn, couples the complex to the actin microfilament network of the cell skeleton [15,114,142,173]. A new p120 catenin was recently identified [15]. Its role remains controversial in contrast to the above-mentioned classical catenins but high affinity binding of p120 catenin to Ve-cadherin suggest that it may regulate vascular permeability and affect BBB function [15, 69, 114]. 

## BBB STRUCTURE: THE CYTOSKELETON

The brain endothelial cytoskeleton has a critical role in establishing interendothelial junctional integrity. The cytoskeleton is composed of three primary elements: actin microfilaments, intermediate filaments and microtubules [68, 85,156,182]. The actin microfilament system is focally linked to multiple membrane adhesion proteins including cadherin or occludin, glycocalix components, functional intercellular proteins like zona occludens (ZO), catenins and focal adhesion complexes [85,167,182]. Short radial bands of F-actin: TJ and AJ actin-associated proteins are assembled to form a structure denoted as the actin-rich adhesion belt [68,156,182]. Actin structure is intimately involved in endothelial cells tension force generated *via* myosin light chain phosphorylation and actin stress fiber formation [78,151]. 

A second major element of the cytoskeletal structure of the brain endothelial cell is the microtubule system. Polymers of α- and β-tubulin form a lattice network of rigid hollow rods that span the cells in a polarized fashion from the nucleus to the periphery. Microtubules participate in rapid assembly of actin filaments and focal adhesion, isometric cellular contraction and/or increased transendothelial leukocyte migration. Some recent studies suggest that these functions are realized *via* interactions of microtubules with microfilaments [71,172]. 

Intermediate filaments, of which vimentin in the major protein in endothelial cells, are the third element involved in the cytoskeletal structure. The possible role of these filaments in cytoskeletal changes is still unclear although some recent studies indicate dynamic changes in vimentin during the reorganization of actin filaments and microtubules [85]. 

## BBB FUNCTION: VESICULAR SYSTEM, ENZYMATIC BARRIER AND TRANSPORT 

Before discussing how BBB junctions are regulated and affected by disease, it is necessary to put those junctions into the context of other BBB properties. These include the general paucity of vesicular transport at the BBB, the presence of enzymes that by degradation prevent the entry of a variety of compounds, and the presence of a wide range of transport systems. Because non-lipid soluble compounds only diffuse slowly across the BBB, the latter are necessary for both the entrance of nutrients into brain and the clearance of waste products from brain.

The cytoplasm of brain endothelial cell is of uniform thickness, with very few pinocytotic vesicles and a lack of fenestrations. The wall thickness of brain capillaries is approximately 40% of that in other types of endothelial cell [37]. It is speculated that this decrease in wall thickness could be an adapation to the restrictive permeability of the BBB, allowing nutrients a shortened transport time to cross through the membrane and cytoplasm, and enter the brain parenchyma [37].

In general, brain endothelial cells have a very low number of vesicles under normal conditions compared to other types of endothelial cells. However, during some disease states (e.g. inflammatory conditions) the number of vesicles can increase. The classic type of caveole (Ω-shaped cell surface invagination, open or close) is one of the most predominant forms of vesicles [34, 97, 98, 154, 166]. Fusion between vesicles may eventually lead to the formation of transendothelial channels and/or vesicle/vacuolar organelles (VVO). Transendothelial channels correspond to chains of two or more fused vesicles that are open simultaneously on the luminal and abluminal side of endothelial cells [34,97,154, 166]. Besides that, channels made by one of the vesicle open on the both sides of endothelial cells can occur. VVO on the other hand are large collection of interconnected vesicles and vacuoles [98,166]. Fusion of vesicles, vacuole with luminal and abluminal plasma membranes create transcellular pathways confirmed in the several ultrastructural studies [29,37,98]. Brain endothelial cells also possess a well developed tubular system formed by membrane-bound tubules that intrude deeply into the endothelial cells from both the luminal and abluminal poles. The tubular system also opens at the level of the lateral intercellular space and branches off in different directions forming an intracellular network resembling a tree-like structure. This network is involved in the transport of circulating proteins and could be involved in transendothelial leukocyte migration [29,37,97].

The BBB is an *enzymatic barrier*, capable of metabolizing drug and nutrients [25,104]. These enzymes are principally directed at metabolizing neuroactive blood-borne solutes. Enzymes such as γ-glutamyl transpeptidase (γ-GTP), alkaline phosphatase, and aromatic acid decarboxylase are found at elevated concentrations in cerebral microvessels, yet are often in low concentration or absent in non-neuronal capillaries. These enzymes are often polarized between the luminal and abluminal membrane surface of brain endothelial cells. This concept is supported from several quantitative biochemical studies [18]. The enzymes γ-GTP and alkaline phosphatases are presented at the luminal endothelium [19].

The BBB possesses a wide array of transporters. Because of the occluded paracellular pathway, nutrients must cross the endothelial cell to gain access to brain from blood. Thus, for example, the BBB has very high levels of the glucose transporter GLUT1, and the large neutral amino acid transporter, LAT1, that facilitate movement of those nutrients from blood to brain [20,21]. There are also efflux transporters that move compounds from brain to blood. These transporters, such as P-glycoprotein and organic anion transporters, are involved in clearing waste products from the brain or preventing the entry of potentially neurotoxic compounds from blood to brain [41,94]. Other transporters are involved in ion homeostasis and the transport of signaling molecules between blood and brain. Many of these transporters have a polarized distribution at the cerebral endothelium. For example, Na^+^-K^+^-ATPase and the sodium-dependent neutral amino acid transporter (A-system) are associated with the abluminal portion of the endothelium [19]. Such structural, pharmacological and biochemical evidence for luminal and abluminal polarization of receptors, enzymes, and channels at the cerebral endothelium established the BBB to be a working, non-stagnant, membrane unequivocally evolved to maintain brain homeostasis. 

Besides transporters, brain endothelial cells posses several ion channels, which control important endothelial functions. Ion channels are involved in the production and release of vasoactive factors (nitric oxide and prostacyclin), trafficking and secretion of haemostatic factors (von Willebrand, tissue type plasminogen activator) and enhancing flux of Ca^2+^ [46,81].

Ions or small molecules like amino acids and glucose are mostly transferred through the brain endothelial cells by transporters/carriers/channels present on the plasma membrane. Most macromolecules move across brain capillary endothelium by bulk phase non-receptor mediated endocytosis. For example, cationic macromolecules prefer uptake by clathrin coated membranes and pits and are subsequently delivered to and degraded in lysosomes [97]. In contrast, anionic molecules, which include most plasma proteins, undergo fluid internalization by apical caveolae and are shuttled to the basal side [97,115]. In general, pathological increases in BBB permeability are mostly associated with increased paracellular permeability although changes in transcellular flux may contribute. 

## PHYSIOLOGY OF THE BBB: TRANSPORT THROUGH THE BBB (PARACELLULAR AND TRANSCELLULAR)

These trans- and paracellular pathways differ with respect to physical properties: a) the transport across the transcellular pathway can be either passive or active while passage trough paracellular route is exclusive passive and it is driven by electrochemical, hydrostatic and osmotic gradients, b) compared to the transcellular route, the paracellular pathway is characterized by higher conductance and lower selectivity; c) paracellular transport is not rectified with the similar conductance and selectivity in either apical to basal or basal to apical directions; d) paracelluar pathways have well defined values of electrical conductance as well as charge and size selectivity [14].

The paracellular permeability of the BBB is maintained by equilibrium between the contractile force generated at the endothelial cytoskeleton and adhesive forces produced at endothelial cell-cell junctions and cell-matrix contacts [58, 176]. A dynamic interaction among these structural elements controls the opening and closing the paracellular pathway and thus serves as a fundamental mechanism in regulation of the blood-brain exchange [58]. The unperturbed endothelial barrier has restrictive properties that are due primarily to the closed junctional complex. Factors which increase paracellular permeability act on the junctional complex resulting in the formation of minute intercellular gaps which can allow the passage of plasma proteins (e.g. albumin), fluid and leukocytes across the barrier. The processes by which interendothelial gaps are formed are the subject of intense investigation. There are two ongoing and probably simultaneous processes: changes in adhesive properties of TJ and AJ proteins and reorganization of the actin cytoskeleton. 

The changes in adhesive property of TJ and AJ proteins are mostly correlated with alterations in their phosphorylation state. In general, the TJ proteins (e.g. occludin, ZO-1, ZO-2, and claudin-5) are phosphoproteins and changes in phosphorylation state affect their interaction, alter transmembrane protein localization and induce their redistribution [65,77,95,160,162]. Phosphorylation of TJ proteins can occur on amino acid residues Ser-, Thr-, and Tyr- although the exact position/sites of phosphorylation in the brain endothelial cells is still unknown. Analyzing published studies about the phosphorylation pattern of TJ and AJ protein in brain endothelial cells, it appears that type of phosphorylation mostly depends on the type of stimulii as well as the local microenvironment. For example, vascular endothelial growth factor and CCL2 induce Ser/Thr phosphorylation and redistribution of occludin and ZO-1 in murine brain endothelial cells, and oxygen mediators induce primarily Tyr-phospho-rylation and reorganization of the TJ complex [65,67,77, 162]. However, in conditions like calcium depletion, phorbol ester treatment or bacterial infection, TJ proteins (occludin) undergo dephosphorylation during TJ disruption rather than additional phoshorylation [33,91,113]. Similar to TJ proteins, AJ proteins like Ve-cadherin and β-catenin undergo phosphorylation of Ser/Thr and Tyr residues during opening of brain endothelial barrier [82,134,188]. 

Phosphorylated TJ and AJ proteins undergo redistribution, a critical event for the changes of adhesive contact between brain endothelial cells. A complete redistribution of TJ proteins can be seen at the time of lowest transendothelial electrical resistance and highest permeability coefficients for the tracers such as FITC-albumin, inulin and mannitol [162,163]. However there is still not firm evidence on how redistribution occurs. Simultaneous, and closely associated with junction protein modification, there is actin cytoskeleton reorganization [16,163]. Actin filament polymerization, actin myosin association and generation of intraendothelial contractile forces, results in “pulling in” of the contact surface between brain endothelial cells which may also result in the dislocation of transmembrane tight and adherence junction proteins [158].

## FACTORS INVOLVED IN PARACELLULAR AND TRANSCELLULAR BRAIN ENDOTHELIAL BARRIER “OPENING”

Depending on the individual pathology, a variety of the factors are involved in altering BBB permeability. Several groups of mediators appear to have a prominent role in BBB disruption. These include a group of *vasogenic agents* including histamine, substance P, endothelin-1 and bradikinin; growth factors such as vascular endothelial growth factor (VEGF), basic fibroblast growth factor bFGF and transforming growth factor–β (TGFβ); a heterogenic group of inflammatory mediators including cytokines [interleukin-1β (IL-1β), tumor necrosis factors-α (TNF-α), interferon-γ (INF-γ )] and chemokines (CCL2 and CXCL8); matrix metalloproteinases (MMP2 and MMP9); free radicals such as O_2_^-^, H_2_O_2_, OH^-^ NOO^-^ and lipid mediators including prostaglandin E2 and F2a [1,6,43,45,99,115,127,136,137,164,190]. Potent inducers of BBB hyperpermeability are also thrombin, amyloid-β, intracellular Ca^2+^ and blood-borne cells like leukocytes where direct interaction with brain endothelial cells causes BBB “opening” [2,23,88,100]. 

Infective agents may also cause BBB disruption. The interactions of these agents with brain endothelial cells is crucial in the pathogenesis of meningitis, and encephalitis particularly in BBB “opening” and their passage into brain parenchyma. Bacteria and bacterial toxin (*Escherichia coli*, *Citrobacter freundii*, *Streptococcus pneumoniae* and lypopolysaccarides, *Cholera* toxin, pertussin toxin, respectively), viruses and virus components (HIV-1, Measles virus-NP), parasites and fungal pathogens not only penetrate through the BBB, they also contribute to its breakdown [79,123,131,171]. Microorganisms appear to be adapted and breached the barrier either by targeting junctions or cells. *Chlamydia* pneumonia exposure to brain capillary endothelia decreases occludin expression while increasing expression of cell-adhesion proteins [101]. The fungal pathogen *Cryptococcus neoformans*, which causes meningitis, alters subcellular occludin localization in human brain capillaries [31].

Despite intense investigation, there are still numerous controversies over how most of these factors affect the BBB. Some of the factors appear to exclusively affect the paracellular permeability (e.g. IL-1β and CXCL8), while some others predominantly act to increase transcellular permeability (e.g. TNF-α) [1]. In addition, the duration of BBB disruption may differ between mediators. Some mediators, e.g. histamine, cause a rapid and transient opening of the BBB related to a fast cytoplasmic accumulation of Ca^2+^, while others, e.g. thrombin, cause prolonged opening of BBB related to robust changes in the endothelial cytoskeleton [176].

## SIGNAL TRANSDUCTION PATHWAYS REGULATING PARACELLULAR AND TRANSCELLULAR PERMEABILITY

The regulation of paracellular permeability involves complex interactions between several agonist-activated signaling pathways and key structural components of the endothelial cells. The latter include, but are not limited to, TJ proteins. One of the most extensively studied regulators of TJ and TJ-mediated permeability is protein kinase C (PKC). TJ proteins are phosphoproteins which possess several phosphorylation sites which could be affected during the brain endothelial TJ complex disassembly. PKCs are a family of serine/threonine kinases that regulate a variety of cell functions including proliferation, gene expression, cell cycle, differentiation, cytoskeletal organization, cell migration and apoptosis [133]. The PKC family includes isozymes (PKC-α, ßI, ßII, γ, δ,  ε ,  η ,  μ ,  θ  , λ  , ζ  ,  τ ,) which are involved in signal transduction from membrane receptors to the nucleus [122,133]. Some of the PKC isoforms are denoted as critical molecules in phosphorylation of TJ proteins. For example, activation of three PKC isoforms (PKC-α/βII, PKC (pan)-βII and PKC-ζ/λ) by viral gp120 leads to cytoskeleton alterations and increased monocyte migration [67,79,162]. Under hypoxic and post-hypoxic reoxygenation conditions and under the influence of endothelin-1, PKC-βII, PKC-γ, PKC- η, PKC-µ and PKCλ regulate TJ disassembly [51,83,126]. Or CCL2 activation PKC isoforms PKCα and PKCζ induced phosphorylation of TJ proteins (occludin, claudin-5, ZO-1 and ZO-2) and brain endothelial barrier hyperpermeability [162]. However, it is very important to note that the activation of PKC isoforms is dependent on the type of activator as well as the type of the endothelial cells. Some PKC isoforms can have opposite roles in different types of endothelial cell. For example, in retinal endothelial cells, VEGF *via* PKCβII induces phosphorylation of occludin and increases permeability, but in brain endothelial cells this PKC isoform plays a protective role in VEGF-induced hyperpermeability [67, 161]. Therefore, it is critical to precisely define when and which type of PKC isoform is activated in brain endothelial cells under different conditions.

Besides serine/threonine residues, phosphorylation of tyrosine residues on TJ and AJ proteins also has a significant role in brain endothelial barrier disassembly. Protein tyrosine kinases (PTKs) are enzymes which catalyze the phosphorylation of tyrosine residues. There are two main classes of PTKs: receptor PTKs and cellular, or non-receptor, PTKs [120]. In regulation of brain endothelial barrier permeability, both classes of PTK play an important role. Receptor PTKs, which have extracellular domains with one or more identifiable structural motifs, have binding sites for several growth factors including EGFR, Eph, FGF, PDGF or VEGF, which are involved in BBB hyperpermeability [12,28,120]. For example, VEGF *via* receptor Flt-1 and its downstream signaling molecules like phosphatidylinositol 3-kinase/Akt (PI3-K/Akt), nitric oxide syntheses (NOS) and protein kinase G (PKG) can cause brain endothelial hyperpermeability and brain edema formation [41,181]. On the other hand, VEGF action *via* flk-1/KDR could induce specific Tyr-phosphorylation of the endothelial adherens junction components VE-cadherin, β-catenin, plakoglobin, and p120 [150]. Similar effects occur with vascular permeable factor (VPF) which *via* Flk-1 (VEGF-R2) and activation of membrane-associated kinases, such as Src and PI3K, induces increased BBB permeability and the development of local edema [150]. It is important to note that activation of PTK can also trigger activation and interaction with some other signaling molecules like nitric oxide (NO), ERK1/2, and PKC in relation to vascular permeability regulation.

In contrast to receptor PTKs, cellular PTKs are located in the cytoplasm, nucleus or anchored to the inner leaflet of the plasma membrane. They are grouped into eight families: Src, JAK, ABL, FAK, FPS, CSK, SYK and BTK [12,28,120]. Each family consists of several members. With the exception of homologous kinase domains (Src Homology 1, or SH1 domains), and some protein-protein interaction domains (SH2 and SH3 domains), they have little structural similarity [120]. The cellular PTKs are involved in cell growth, cell differentiation, and/or cell adhesion. Some members of this family (e.g. JAKs), have a role in phosphorylation of STAT transcription factors [54,120]. Reactive oxygen species are thought to primarily act through these kinases. For instance, reactive oxygen species generated during ischemia, brain injury, monocytes/neutrophil activation or alcohol exposure can activate matrix metalloproteinases (MMP-1, -2, and -9) and decrease tissue inhibitors of MMPs (TIMP-1 and -2) in a PTK-dependent manner [65,66]. The increase in MMPs and PTK activation is associated with degradation of endothelial basement membrane and enhanced tyrosine phosphorylation of TJ protein. Such changes may result in increased permeability and monocyte migration in stroke, in HIV-1 encephalitis, or in multiple sclerosis [20,140,170]. Pretreatment with PP1 could improve outcomes in the most of these conditions and strongly suggests a possible role of Src tyrosine kinase in BBB permeability regulation [65,66]. 

Mediators of oxidative stress also regulate brain endothelial permeability *via* a complex system of MAP kinases (p38, ERK1/2). MAP kinase could be a “nodal point” in, for example, PTK signaling [52]. However, it may also be a direct executor of TJ protein phosphorylation in conditions like exposure to 4-hydroxy-2-nonenal (4-HNE), one of the major biologically active aldehydes formed during inflammation and oxidative stress [174]. Human immunodeficiency virus-1 (HIV-1) Tat protein exerts similar effects, regulating expression of claudin-5 mRNA in brain endothelial cells *via* ERK1/2 activation but simultaneous activation of ERK1/2, PI-3K, and nuclear factor-κB (NF-κB) mediated alterations and distribution of claudin-5 protein levels in Tat treated brain endothelial cells [3]. Chronic exposure to alcohol with simultaneous treatment with lipopolysaccharide (an inflammatory mediator) also triggers brain endothelial barrier “opening” and TJ protein phosphorylation *via* activation of ERK 1/2 and p38 kinase as well as Jun-N-terminal Kinase (JNK) and activation of NFκB RelA-p50 [155]. 

In recent years, significant attention has focused on the family of small RhoGTPases as major regulators of TJ formation, maintenance and disruption. Rho, GEF-H1 a guanine nucleotide exchange factor for Rho, Rac, CdC42 and ROCK are some of the member of RhoGTPase family [75]. They are known to mediate cytoskeletal contractile responses *via* myosin ATPase activity. For example, ROCK promotes phosphorylation of the regulatory light-chain of myosin (MLC) on Ser19 and Thr18 through phosphorylation of the myosin light-chain phosphatase (MLCP) and *via* blocking of MLC dephosphorylation. This site-specific phosphorylation of MLC in turn elevates myosin ATPase activity, leading to actin-myosin contraction [116,118]. This pathway has been described as a mechanism underlying “long-lasting” alterations in endothelial permeability caused by thrombin [176]. Some other factors, such as TGF-β, C5a-activated neutrophils, HIV infected monocytes, CCL2, ICAM-1, *Cryptococcus neoformans*, histamine and VEGF may also utilize this pathway [30,48,130,163,179]. Besides inducing specific actin filament polymerization (stress fiber formation) which in turn generates contractile intraendothelial forces, RhoGTPase family members (Rho, ROCK) also induce phosphorylation of TJ and AJ proteins and their redistribution [26]. Two possibilities of how Rho and ROCK act are direct action on TJ and AJ complexes and indirect action *via* downstream activation of other signaling molecules (e.g. PKCα) [162].

The signaling pathways involved in regulating BBB permeability are still the subject of the very intensive investigation. Much current investigation is focused on defining the role of novel multiple signal pathways associated with BBB “opening” and how they interact at multiple levels. This is important in determining how signals are integrated and affect the phosphorylation of junction proteins and/or actin cytoskeletal remodeling. 

## LESSONS FROM BBB PATHOLOGY

Disruption of the BBB is generally believed to be harmful in most circumstances as it can cause the influx of leukocytes, potentially neuroactive compounds and water (edema) from blood. Many CNS diseases, including a diverse range of inflammatory diseases, diabetes, cancer, and microbial infection, cause such disruption. More often than not, this reflects a change in the TJ itself. 

The most progressive BBB breakdown is associated with a diverse range of CNS inflammatory conditions. Whether the inflammation is primary, resulting from infection (meningitis, meningoencepahlits, encephalitis HIV infections) or an autoimmune disorder (multiple sclerosis), or secondary (as occurs following stroke or brain trauma), BBB breakdown is associated with several changes in brain endothelial phenotype (proinflammatory phenotype), junctional complex remodeling and a progressive increase in leukocyte infiltration [2,44,79,82,124,128,131,145,171]. Under some circumstances (stroke and brain trauma), vasogenic brain edema develops as a result of junction disruption [44,145]. A variety of inflammatory mediators participate in these pathological changes in brain endothelial junctional complexes. Different cytokines (IL-1β, TNF-α, INF-γ), chemokines (CXCL8, CCL2) matrix metalloproteinases (MMP-2, MMP9) and adhesion molecules (ICAM-1) play a role, which may be direct or indirect, *via* attraction of leukocytes [6,43,45,99,105,115, 127,137,164,190]. 

Neurodegenerative disease like Alzheimer or Parkinson disease may also display changes in BBB permeability [42,62]. Clinical and experimental studies show that BBB impairment is closely associated with increased rates of neurodegeneration in patients with Alzheimer disease and transgenic mice overexpressing APP695 in Tg2576 mice [62,87]. Increased BBB permeability is indeed hypothesized as a potential mechanism by which vascular β-amyloid accumulates in the brain parenchyma [23,87]. Transient BBB opening occurs in Parkinson’s disease, although most impairment occurs at the blood-cerebrospinal fluid barrier [42]. Again, such disruption is associated with transient secretion of inflammatory mediators.

Transient “opening” of BBB is also present before, during and after epileptic seizures. There a multiple factors involved in etiology of BBB impartment in epilepsy, including inflammatory mediators (epilepsy associated with stroke or brain trauma) and metabolic disorders [102].

Either primary or secondary (metastasis in the brain) brain tumors can induce BBB disruption. This is associated with disturbance of TJ complex which is manifested as a loss of staining for claudin-5, occludin and ZO-1. In addition, newly formed blood vessels in gliomas have abnormal TJ complexes with very low expression of some critical TJ proteins [93,145]. Alterations in BBB permeability are thought to be caused by accumulation of growth factors (VEGF or HGF) as well as proinflammtory cytokines [145]. One consequence of BBB disruption in brain tumors is the development of vasogenic edema which may be fatal.

Diabetes types II, hepatic encephalopathy (caused by hyperammonemia), or encephalopathy linked to thiamine deficiency are some of the metabolic disorders associated with BBB disturbance [149,170,183]. Accumulation of oxidative mediators, cytokines or matrix metalloproteinase MMP-9 are some of the factors that may be involved in BBB disruption in these conditions [72,76]. 

What lessons can be drawn from these neuropathological conditions? One common underlying factor for all of these neuropathological conditions is that inflammation and inflammatory mediators have a critical role in BBB disruption. These inflammatory factors mostly act focally/locally to induce paracellular opening (TJ complex remodeling). Inflammatory mediators and the process of inflammation may, therefore, be a good target for controlling BBB “opening” and “closing”.

## NEUROPHAMACOLOGICAL SIGNIFICANCE OF BBB “OPENING”

There are two major reasons why is important to understand the mechanism underlying BBB “opening” and to be able to control such disruption. First, uncontrolled BBB “opening” may damage the brain parenchyma by enhancing leukocyte influx and vasogenic edema (e.g. in stroke, brain trauma or multiple sclerosis). Second, the normal BBB restricts the entry of potential therapeutic agents into brain. This, in large portion, has limited successful treatment of many severe CNS conditions such as brain tumors and neurodegenerative diseases [185]. The ability to modulate BBB permeability would enable physicians to prevent the adverse effects of BBB disruption in disease states or to enhance BBB permeability to permit the efficient transfer of drugs to brain. 

Currently, the only therapeutic agents in use that improve BBB integrity are steroids [185]. They can have a marked effect on edema formation in tumors but are not effective in stroke. The precise mechanisms by which steroids (dexamethasone, hydrocortisone) affect the BBB are still uncertain. However, several *in vitro* and *in vivo* studies indicate that steroid therapy promotes BBB integrity through anti-inflammatory actions (decreased cytokine production and NFκB activation) as well as by stabilizing the TJ complex [139,184]. Some *in vitro* studies have indicated that hydrocortisone increases occludin mRNA and protein by acting on the occludin promoter region *via* the glucocorticoid receptor [139,184]. 

Inflammatory mediators appear to play a role in BBB disruption in many disease states and, therefore, inhibition of cytokines, chemokines or adhesion molecules is a potential therapeutic target [57]. Unfortunately, the use of antibodies to target cytokines and adhesion molecules in man has so far failed because of unwanted side effects [57]. There have been some promising results with chemokine inhibition, due to the fact that small peptide chemokine inhibitors display much less side effect. 

Another potential therapeutic strategy is directed at stabilizing brain endothelial cell:cell interactions and the junctional complex. For example, S1p or SSeCKS mediators appear to decrease BBB permeability by acting primarily on TJ proteins [89,90]. Their activity is still the subject of intense investigation and it will be very interesting to see which signal molecules are involved in their actions on TJ proteins.

Another strategy, which is in the recent years has been gaining more attention, is targeting the signals molecules involved in remodeling junctional complexes. For example, targeting Rho, Rho kinase, MLCK, PTKs or PKC isoforms could be a promising strategy. RhoGTPases have significant effects on BBB integrity and the inflammatory response. They act as “on-and off-switches” and have a fundamental importance for increased permeability as well as in the time-dependent restoration of endothelial barrier function. Selective RhoGTPase inhibitors have produced promising results in some of the vascular as well as cerebrovascular disorders [30,182]. 

## OPENING THE BBB FOR DRUG DELIVERY

In conditions such as brain tumors, neurodegenerative disorders like Alzheimer’s disease, Parkinson’s disease or different types of metabolic disorders with developing encephalopathy, therapeutic strategies are often limited by the inability of drugs to cross the BBB from blood to brain. There has, therefore, been much interest in devising methods for disrupting the BBB. The ultimate goals of such methods are several: (i) there should be specificity in targeting the brain or preferably the diseased/injured part of the brain; (ii) the action should be brief in duration with the paracellular opening reverting back to control quickly; and (iii) the paracellular leak should be specific for the class of molecules one wishes to deliver. 

There are several options for transiently disrupting the BBB. The most widely studied method involves intra-arterial injection of a hyperosmolar osmotic such as mannitol, which leads to endothelial cell shrinkage and opening of TJ. This method has been used extensively to deliver chemotherapeutic agents to gliomas, neuroectodermal tumors, CNS lymphomas, and brain metastases in animal models and patients by Neuwelt and colleagues [63,84,152]. Some specificity in targeting is achieved by injecting the hyperosmotic agent unilaterally into the cerebral circulation. Further data is still required before this technique gains widespread use. 

Bradikinin and its analog RPM-7 (B2 receptor agonist) have also been investigated as potential inducers of transient BBB disruption [13,135]. Despite its longer half-life and greater selectivity, the use of RMP-7 in conjunction with chemotherapy showed no benefit in a randomized phase 2 trials for gliomas. 

Other potential methods to induce transient “opening” of BBB are intra-arterial administration of alkilglycerola, which transiently increases the penetration of drugs or macromolecules across the BBB, and exposure of BBB to radiotherapy (20-30Gy) whilst chemotherapy is administered [47,84,138]. These strategies are under intense investigation in order to understand their mechanisms of action at the BBB and possible side effects. 

A promising target to induce transient opening of the BBB for drug delivery may also be the signaling molecules involved in regulation of BBB permeability. We would like to point out that effect of Rho and Rho kinase in transient opening of brain endothelial barrier should be take in consideration for the further investigation of drugs deliver into brain.

An alternative strategy for drug delivery across the BBB focuses on transcytosis and transcellular permeability. Enhanced vesicular transport can be used for delivery compounds into the brain. Examples are: liposome-born therapeutic agents that show more transendothelial passage under hypothermic conditions and compounds which bind to lectins and *via* adsorptive vesicular transport are taken up into brain parenchyma [10,11,105,189]. Modulation of specific endothelial carrier proteins (transporters) is also a therapeutic target for enhancing drug delivery across the BBB. Such transporters may be involved in transport of compounds from blood to brain, in which case drugs which are substrates will have enhanced delivery (e.g. L-DOPA is a substrate for the L-system amino acid transporter). There are, however, also efflux transporters which clear compounds from the brain. P-glycoprotein (P-gp) is the major BBB efflux transporter and mice that lack P-gp have higher brain uptake of a wide range of drugs [96,180]. There has, therefore, been a major effort by drug companies to develop P-gp inhibitors to enhance the delivery of drugs to the brain. As yet, there have been no clinical trials that have shown a benefit of this approach to enhance drug delivery to brain.

From clinical and neuropathological point of view, controlling BBB permeability has tremendous importance in treatment of the devastating brain disorders. From the neuropharmacological point of view controlling BBB permeability should be effective, and without or with limited side effects. Thus, understanding BBB structure and the molecular mechanisms of regulating BBB permeability opens a new avenue of therapeutic strategies for most brain disorders.

## CONCLUSION 

Much exciting progress is being made in understanding the molecular basis of BBB paracellular permeability. Although some details remain obscure, the TJ is likely to be site where multiple cellular signaling pathways converge to regulate paracellular permeability. Due to this fact, further intensive investigation is needed to completely understand the mechanism of regulating permeability, but they promise to make important findings for the treatment of severe CNS diseases such as multiple sclerosis, stroke, infections and brain tumors.

## Figures and Tables

**Table 1:** Proteins Identified in Brain Endothelial Junctional Complex